# Dietary Determinants of Changes in Waist Circumference Adjusted for Body Mass Index – a Proxy Measure of Visceral Adiposity

**DOI:** 10.1371/journal.pone.0011588

**Published:** 2010-07-14

**Authors:** Dora Romaguera, Lars Ängquist, Huaidong Du, Marianne Uhre Jakobsen, Nita G. Forouhi, Jytte Halkjær, Edith J. M. Feskens, Daphne L. van der A, Giovanna Masala, Annika Steffen, Domenico Palli, Nicholas J. Wareham, Kim Overvad, Anne Tjønneland, Heiner Boeing, Elio Riboli, Thorkild I. A. Sørensen

**Affiliations:** 1 Department of Epidemiology and Biostatistics, School of Public Health, Imperial College London, London, United Kingdom; 2 Institute of Preventive Medicine, Copenhagen University Hospital, Copenhagen, Denmark; 3 National Institute for Public Health and the Environment (RIVM), Bilthoven, The Netherlands; 4 Department of Human Biology, Nutrition and Toxicology Research Institute of Maastricht (NUTRIM), Maastricht, The Netherlands; 5 Department of Clinical Epidemiology, Aarhus University Hospital, Aalborg, Denmark; 6 MRC Epidemiology Unit, Institute of Metabolic Science, Addenbrooke's Hospital, Cambridge, United Kingdom; 7 Danish Cancer Society, Institute of Cancer Epidemiology, Copenhagen, Denmark; 8 Division of Human Nutrition, Wageningen University, Wageningen, The Netherlands; 9 Molecular and Nutritional Epidemiology Unit, ISPO (Cancer Research and Prevention Institute), Florence, Italy; 10 Department of Epidemiology, German Institute of Human Nutrition, Potsdam-Rehbruecke, Nuthetal, Germany; 11 Department of Epidemiology, School of Public Health, Aarhus University, Aarhus, Denmark; 12 Department of Cardiology, Aalborg Hospital, Aarhus University Hospital, Aalborg, Denmark; University of Las Palmas de Gran Canaria, Spain

## Abstract

**Background:**

Given the recognized health effects of visceral fat, the understanding of how diet can modulate changes in the phenotype “waist circumference for a given body mass index (WC_BMI_)”, a proxy measure of visceral adiposity, is deemed necessary. Hence, the objective of the present study was to assess the association between dietary factors and prospective changes in visceral adiposity as measured by changes in the phenotype WC_BMI_.

**Methods and Findings:**

We analyzed data from 48,631 men and women from 5 countries participating in the European Prospective Investigation into Cancer and Nutrition (EPIC) study. Anthropometric measurements were obtained at baseline and after a median follow-up time of 5.5 years. WC_BMI_ was defined as the residuals of waist circumference regressed on body mass index, and annual change in WC_BMI_ (ΔWC_BMI_, cm/y) was defined as the difference between residuals at follow-up and baseline, divided by follow-up time. The association between energy, energy density (ED), macronutrients, alcohol, glycemic index (GI), glycemic load (GL), fibre and ΔWC_BMI_ was modelled using centre-specific adjusted linear regression, and random-effects meta-analyses to obtain pooled estimates. Men and women with higher ED and GI diets showed significant increases in their WC_BMI_, compared to those with lower ED and GI [1 kcal/g greater ED predicted a ΔWC_BMI_ of 0.09 cm (95% CI 0.05 to 0.13) in men and 0.15 cm (95% CI 0.09 to 0.21) in women; 10 units greater GI predicted a ΔWC_BMI_ of 0.07 cm (95% CI 0.03 to 0.12) in men and 0.06 cm (95% CI 0.03 to 0.10) in women]. Among women, lower fibre intake, higher GL, and higher alcohol consumption also predicted a higher ΔWC_BMI_.

**Conclusions:**

Results of this study suggest that a diet with low GI and ED may prevent visceral adiposity, defined as the prospective changes in WC_BMI_. Additional effects may be obtained among women of low alcohol, low GL, and high fibre intake.

## Introduction

There is a wealth of evidence indicating that waist circumference (WC) remains a significant predictor of chronic diseases such as type 2 diabetes, cardiovascular diseases, cancer, as well as total mortality after adjusting for body mass index (BMI) or fat mass [1], [2], [3], [4], [5], [6], [7], [8]. According to imaging studies, the phenotype “WC for a given BMI (WC_BMI_)” can be considered a good proxy measure of visceral adiposity [9], [10], which thus seems to encompass the major disease risk associated with obesity.

Given the widely recognized effects of WC_BMI_ on health, the understanding of how diet can modulate changes in this phenotype is deemed necessary. Despite the fact that the evidence from epidemiological observational studies linking diet to obesity is at most inconclusive [11], it has been suggested that some aspects of the diet may influence body fat distribution [12], [13]. However, most of previous studies were based on measurements of WC, a marker of abdominal adiposity which is highly correlated with BMI. Hence, the objective of the present study was to assess the association between dietary factors and prospective changes in visceral adiposity as measured by the phenotype WC_BMI_, i.e. changes of WC that are independent of changes in BMI.

## Methods

### Ethics Statement

The European Prospective Investigation into Cancer and Nutrition (EPIC) study has been approved by local review board of all participating institutions. Written informed consent was obtained from all participants before joining the EPIC study.

### Participants

The current study included participants from eight centres in five countries involved in the EPIC study, participating in the DiOGenes (Diet, Obesity and Genes) project, namely Florence (Italy), Norfolk (UK), Amsterdam, Maastricht and Doetinchem (the Netherlands), Potsdam (Germany), Copenhagen and Aarhus (Denmark). Detailed information on the study population and data collection of the EPIC study has been described elsewhere [14]. At baseline (between1992–1998), participants filled out extensive questionnaires covering their diet, lifestyle, and medical history, and anthropometric measurements were obtained. Updated information on anthropometric data has been obtained from EPIC participants through follow-up examinations during 1998–2005 (median follow-up time 5.5 years). Of the 146,543 participants at baseline, 102,346 participated in the follow-up examination (69.8% response rate). Given that the current study forms part of larger project aiming at looking at gene*diet interactions in the development of visceral adiposity, we excluded individuals with no blood samples collected (n = 4,048). We also excluded pregnant women (n = 133), those with missing information on diet or anthropometrics (n = 1,266), those in the lowest and highest 1% of the EPIC cohort distribution of the ratio of reported total energy intake: energy requirement (n = 752), individuals with prevalent chronic diseases (cancer, diabetes and/or cardiovascular disease) at baseline (n = 3,811) and incident chronic diseases during follow-up (n = 5,132), and those with unrealistic anthropometric measurements (n = 115). In order to avoid the variable changes in body composition and shape in old age and the possible underlying subclinical disease processes that occur with age, we excluded from the analyses participants with age at baseline >60 years or age at follow-up >65 years (n = 31,645). Finally, given the recognized effect of smoking and changes in smoking status on body weight and waist gain, those with missing information on smoking or changing smoking status between baseline and follow-up (n = 7,163) were excluded. In total 48,631 participants (19,694 men and 28,937 women) were included in the analyses (5,081 from Italy; 6,266 from the UK; 6,477 from the Netherlands; 8,661 from Germany; and 22,146 from Denmark).

### Dietary assessment

Usual food intakes were measured using country-specific validated food frequency questionnaires [15]. Individual energy and nutrient intakes – including protein, carbohydrates, fat, alcohol, and fibre – were derived using food composition tables specific to the country [16]. Energy density (ED) was calculated as energy (kcal) from foods (solid foods and semi-solid or liquid foods such as soups) divided by the weights (g) of these foods. It was *a priori* decided not to include drinks (including water, tea, coffee, juice, soft drinks, alcoholic drinks and milk) in the calculation of ED, given that it may dilute the associations of ED with waist circumference [17]. A glycemic index (GI) database was specially developed using mainly published information, under the joint efforts of the EPIC and the DiOGenes projects [18]. Dietary GI was calculated as the weighted average of GI values (GI of glucose = 100) of foods consumed per day. The glycemic load (GL) was calculated as the product of the GI multiplied by the total available carbohydrate intake (g per day), divided by 100.

The exposures of interest in this study were the intake of energy, ED, macronutrients (carbohydrates, proteins, and fats), alcohol, fibre, GI and GL.

### Anthropometric measurements

At baseline, all participants were measured for weight, height and waist circumference. The methods used have been previously described in detail [19]. In brief, body weight and height were measured when participants wore light clothes and no shoes. Waist circumference was measured either at the midway between the lowest rib and the iliac crest (the Netherlands, and Potsdam-Germany) or at the narrowest torso circumference (the other centres). At follow-up examinations, participants in Norfolk (United Kingdom) and Doetinchem (the Netherlands) were measured by trained technicians using the same protocols as at baseline, whereas other centres provided self-reported data. For the latter, guidance was provided to measure waist circumference as at baseline, except for Denmark in which participants were guided to measure their waist circumference at the umbilicus (the reason for changing the site of measurement was to simplify the measurement instructions for participants). Owing to the differences in the methods used to collect anthropometric data at follow-up and the length of follow-up, participants from Doetinchem (the Netherlands) were treated separately from those from Amsterdam and Maastricht (the Netherlands), whereas participants from Copenhagen and Aarhus (Denmark) were combined because no such differences between these two groups existed.

The outcome of interest in the present study was annual change in the phenotype WC_BMI_ (ΔWC_BMI_ in cm/year). This phenotype was defined both at baseline (baseline WC_BMI_) and at follow-up (follow-up WC_BMI_) as the residual values from the gender- and centre-specific regression equations of WC on BMI (using baseline and follow-up WC and BMI values respectively) [20], [21]. Annual changes in this phenotype (ΔWC_BMI_) were calculated as (follow-up WC_BMI_ – baseline WC_BMI_)/follow-up time.

### Assessment of other covariates

Information on age, gender, physical activity, education level, smoking (never, former, and current smoker), menopausal status (premenopausal, perimenopausal, and postmenopausal status), and use of hormone replacement therapy (yes/no or unknown) was collected through self-administered questionnaires at baseline. Physical activity level was indexed into five categories (inactive, moderately inactive, moderately active, active or unknown) based on occupational and recreational activities [22]. Education level was inquired as the highest level of school achieved and participants were classified into primary school and less, technical–professional school, secondary school, university degree, or unknown.

### Statistical analyses

Data were analyzed separately for men and women because it was believed that gender may influence the accumulation of visceral fat. Descriptive analyses showed anthropometric and dietary characteristics of the sample by gender.

The association between dietary variables and ΔWC_BMI_ (in cm/year) was modelled using multi-adjusted linear regression analyses: centre-stratified analyses were carried out first, and random-effect meta-analyses were used to evaluate heterogeneity (*I* ^2^) across study centres, and to obtain pooled estimates of the associations. All analyses were adjusted for baseline age (years), baseline weight (kg), baseline height (cm) and baseline WC_BMI_ (cm), smoking, alcohol intake (except for the model assessing the effect of alcohol intake on WC_BMI_ change: non-drinker, 0.1–4.9 g per day, 4.9–15 g per day, 15–30 g per day, 30–60 g per day and >60 g per day), physical activity, education, and follow-up duration (years). In women, analyses were also adjusted for menopausal status and hormone replacement therapy use.

In the model assessing the association between energy intake and ΔWC_BMI_, total energy was entered as a continuous variable (per 1 kcal increase). The association between ED (per 1 kcal/g increase) and ΔWC_BMI_ was further adjusted for energy derived from drinks.

The intakes of macronutrients and alcohol were adjusted for energy using the nutrient density method; hence, the energy content of each macronutrient/alcohol, expressed as a proportion of total energy (per 5% increase), was included in the model, together with total energy intake. Other methods of energy adjustment were also applied (residual method, multivariate nutrient density method, and energy partition method) yielding very similar results [23]. Therefore only results using the nutrient density method are presented.

The model assessing the association between dietary fibre (included as continuous variable per 10 g increase) and ΔWC_BMI_ was further adjusted for GI (per 10 unit increase), carbohydrates, fat and protein. The same model was used to assess the effect of GI on ΔWC_BMI_ (therefore, fibre and GI were mutually adjusted). The model assessing the effect of GL (per 50 unit increase) on ΔWC_BMI_ was further adjusted for fibre, protein, fat, and total energy. All the dietary variables included in these models (fibre, GI, GL, carbohydrate, fat and protein) were adjusted for total energy intake using the residual method [23].

In sensitivity analyses, we further adjusted all models by misreporting of energy intake. Misreporting of energy intake was estimated using the ratio of reported energy intake to predicted basal metabolic rate (EI:BMR). Subjects were classified as under-reporters (EI:BMR <1.14), plausible reporters (EI:BMR  = 1.14−2.1) or over-reporters (EI:BMR>2.1) of energy intake using cut-off points proposed by Goldberg [24]. However, given that results were virtually unchanged, we decided to presents results without this adjustment. We also checked whether there was an effect modification according to physical activity level (sedentary versus active) and BMI category at baseline (BMI <25 kg.m^−2^ and BMI ≥25 kg.m^−2^), by modelling interaction terms between physical activity/BMI category and dietary factors, and conducting stratified analyses.

All statistical analyses were performed with the STATA statistical package 10.0 (College Station TX).

## Results

The average annual changes in BMI and WC in men were 0.05 (SD 0.25) kg/m^2^ and 0.53 (SD 1.08) cm respectively; the equivalent changes in women were 0.06 (SD 0.31) kg/m^2^ and 0.94 (SD 1.31) cm (data not shown). Information on the anthropometric and dietary characteristics of the sample is shown in **Table 1**.

**Table 1:** Anthropometric and dietary characteristics of the sample.

	Men				Women			
	mean (SD)	p5	p50	p95	mean (SD)	p5	p50	p95
**Baseline age (y)**	50.20 (6.46)	37.84	52.00	58.00	49.15 (7.14)	35.36	51.00	58.00
**Baseline BMI (kg/m^2^)**	26.01 (3.20)	21.28	25.73	31.69	24.88 (3.95)	19.87	24.16	32.34
**Baseline WC (cm)**	93.25 (9.26)	79.00	93.00	109.00	79.13 (10.24)	65.50	77.50	98.50
**Baseline WC_BMI_ (cm)**	0.00 (4.70)	−7.59	−0.03	7.78	0.00 (5.18)	−7.87	−0.30	8.74
**Follow-up BMI (kg/m^2^)**	26.28 (3.33)	21.47	25.95	32.21	25.26 (4.13)	19.95	24.55	33.09
**Follow-up WC (cm)**	96.74 (9.49)	83.00	96.00	113.00	85.29 (11.38)	69.50	84.00	106.00
**Follow-up WC_BMI_ (cm)**	0.00 (5.54)	−8.66	−0.05	8.90	0.00 (6.38)	−9.61	−0.34	11.05
**ΔWC_BMI_ (cm/y)**	−0.01 (0.88)	−1.42	0.00	1.40	0.00 (1.08)	−1.68	−0.03	1.83
**Total Energy (kcal)**	2470 (612)	1568	2409	3578	1970 (511)	1237	1912	2908
**Energy Density (kcal/g)**	1.68 (0.26)	1.27	1.68	2.11	1.51 (0.26)	1.10	1.51	1.95
**Carbohydrates (% E)**	42.03 (6.43)	31.82	41.83	52.99	44.73 (6.31)	34.52	44.69	55.10
**Protein (% E)**	16.37 (2.65)	12.34	16.22	20.94	16.75 (2.80)	12.39	16.63	21.55
**Fat (% E)**	34.94 (5.32)	25.91	35.12	43.28	34.55 (5.48)	25.40	34.67	43.34
**Alcohol (% E)**	6.66 (5.97)	0.22	4.96	18.75	3.98 (4.65)	0.00	2.52	13.67
**Glycemic Index**	58.78 (4.40)	51.86	58.70	66.09	56.61 (4.21)	49.97	56.50	63.66
**Glycemic Load**	134.7 (21.43)	97.31	133.9	175.0	137.4 (19.8)	106.6	136.8	169.9
**Fibre (g)**	22.17 (5.85)	13.48	21.72	32.26	23.64 (5.38)	16.05	23.02	33.25

% E  =  Percentage of total energy intake provided by each nutrient.

**Table 2** shows the multi-adjusted prospective ΔWC_BMI_ associated with the intake of total energy, ED, macronutrients, alcohol, as well as dietary fibre, GI, and GL. No significant association between the intake of total energy and macronutrients (protein, fat, and carbohydrates) and ΔWC_BMI_ was observed, except for a weak inverse association between carbohydrates and ΔWC_BMI_ in women. Alcohol intake was also modestly associated with ΔWC_BMI_ in women only. A diet with higher ED was significantly associated with a greater annual gain in WC_BMI_, in both men and women: 1 kcal/g increase in ED predicted 0.09 cm (95% CI 0.05 to 0.13) and 0.15 cm (95% CI 0.09 to 0.21) higher ΔWC_BMI_ in men and women respectively. No changes in the association between ED and ΔWC_BMI_ were observed when total energy intake was included as a covariate in the model (data not shown). Men and women with higher GI showed higher ΔWC_BMI_ (multi-adjusted ΔWC_BMI_ associated with 10 units increase in GI: 0.07 cm, 95% CI 0.03 to 0.12, in men; 0.06 cm, 95% CI 0.03 to 0.10, in women). Higher GL diets were significantly associated with higher ΔWC_BMI_ in women (50 units increase in GL predicted a 0.09 cm (95% CI 0.01 to 0.17) increase in WC_BMI_); in men, the corresponding increase in WC_BMI_ was 0.05 cm (95% CI -0.02 to 0.13). A high intake of total dietary fibre predicted a significantly lower ΔWC_BMI_ in women but not in men: 10 g increase in fibre intake was associated with −0.06 cm (95% CI -0.08 to -0.03) and −0.01 cm (95% CI -0.03 to 0.01) lower ΔWC_BMI_ in women and men respectively.

**Table 2:** Estimated effect of dietary factors on annual change in “waist circumference for a given body mass index (ΔWC_BMI_, cm/y)”.

		Men			Women	
	β^1^	(95% CI)	*P*	β^1^	(95% CI)	*P*
**Total Energy (kcal)**	−0.00	(−0.00 to 0.00)	*0.40*	0.00	(−0.00 to 0.00)	*0.44*
**Energy Density (kcal/g)** 2	0.09	(0.05 to 0.13)	*<0.001*	0.15	(0.09 to 0.21)*	*<0.001*
**Carbohydrates (5% E)** 3	−0.01	(−0.02 to 0.00)	*0.26*	−0.01	(−0.02 to −0.00)	*0.05*
**Protein (5% E)** 3	−0.02	(−0.06 to 0.03)	*0.54*	−0.03	(−0.06 to 0.01)	*0.13*
**Fat (5% E)** 3	0.01	(−0.00 to 0.02)	*0.06*	0.02	(−0.00 to 0.04)	*0.08*
**Alcohol (5% E)** 3	0.01	(−0.00 to 0.02)	*0.25*	0.02	(0.01 to 0.03)	*0.003*
**Glycemic Index (10 unit)** 4	0.07	(0.03 to 0.12)	*0.002*	0.06	(0.03 to 0.10)	*0.001*
**Glycemic Load (50 units)** 5	0.05	(−0.02 to 0.13)	*0.187*	0.09	(0.01 to 0.17)	*0.030*
**Fibre (10 g)** 6	−0.01	(−0.03 to 0.01)	*0.24*	−0.06	(−0.08 to −0.03)	*<0.001*

% E  =  Percentage of total energy intake provided by each nutrient.

1 The association between nutrient intake and ΔWC_BMI_ was modelled using centre-specific linear regression [adjusting for: age, baseline weight, baseline height, baseline WC_BMI_, smoking, alcohol intake, physical activity, education, follow-up duration, menopausal status (women only), and hormone replacement therapy use (women only)], and random-effect meta-analyses to evaluate heterogeneity (*I* ^2^) across study centres and to obtain pooled estimates of the associations.

*indicates that there is heterogeneity across study centres (*P* for heterogeneity <0.05).

2 further adjusted for energy derived from drinks.

3 further adjusted for total energy.

4 further adjusted for fibre, carbohydrate, fat, and protein.

5 further adjusted for fibre, fat, protein, and total energy.

6 further adjusted for glycemic index, carbohydrate, fat, and protein.

The effect of ED and GI on ΔWC_BMI_ by study centre is shown as supporting information (**Figure S1** and **Figure S2**). There was no evidence of heterogeneity among study centres, except for the association between ED and ΔWC_BMI_ in women (*P* for heterogeneity =  0.035), driven by a single centre (Doetinchem, the Netherlands) with a small sample size and broad confidence interval, that deviated from the other centres. There was no evidence of effect modification in any of the significant associations between dietary factors and ΔWC_BMI_ by physical activity level or baseline BMI category (data not shown).

## Discussion

Results of the present study suggest that some dietary factors, such as ED and GI, may influence the long term accumulation of visceral fat mass, defined as the change in the phenotype “WC for a given BMI (WC_BMI_)”, i.e. changes in the residuals of WC regressed on BMI at two different points in time.

Previous evidence from intervention and observational studies on the dietary determinants of visceral adiposity is limited and conflicting. Most intervention studies were focussed on weight loss and/or total calorie reduction [25], and only a few studies have examined the effect of *ad libitum* dietary interventions on weight and WC change [26]. Overall, results from intervention studies suggest that there is no compelling evidence of a weight loss intervention that targets visceral fat preferentially. On the other hand, some observational studies have suggested that certain dietary factors may modulate body fat distribution [13], [17], [27], [28], [29]; nevertheless, most of previous studies were either cross-sectional, or assessed only the effect of diet on WC changes, highly correlated with BMI, without taking into account concurrent changes in weight or BMI.

Strengths of this study include its prospective design; the possibility of assessing BMI and WC at two time points to calculate changes in WC which are independent of concurrent change in weight or BMI, and hence are likely to represent modifications in the visceral fat depots; the inclusion of a large sample size of adults from different European countries with diversity in their dietary intakes; the application of a strict exclusion criteria so as to eliminate from the sample participants who may have changed their weight or WC as a results of other factors potentially confounding the effects of diet, but difficult to adjust for (i.e. older individuals, those with prevalent and incident chronic diseases, those with unknown smoking status or changes in their smoking status); and the possibility to control for a large number of plausible confounding factors and/or effect modifiers, such as physical activity level. In addition, the lack of centre heterogeneity in the associations between dietary factors and ΔWC_BMI_ reinforces the present findings.

Some limitations should also be considered: the assessment of diet using food frequency questionnaires is subjected to measurement error; in addition, diet was measured only once (at baseline) therefore it was assumed that this assessment was a good proxy measurement of diet during follow up. Misreporting of diet is a major concern in epidemiological studies looking at the association between diet and measurements of body fatness; however, in prospective studies it is less likely that weight-dependent bias in reporting the diet influence the prospective change in body composition, when baseline weight and waist are controlled for; in addition, results were virtually unchanged after adjustment for misreporting of energy. Anthropometric values were self-reported at follow-up in 4 out of the 6 study centres, and this may have introduced some bias due to the selective under-reporting of weight and/or WC among the overweight or the obese[30], [31]. Nevertheless, as results of the meta-analyses show, no obvious differences in associations were observed between centres with different method of anthropometric assessment. Finally, selection bias in the present study may have led to the inclusion of an overall healthier subsample. Indeed, participants in the present study were more likely to be physically active, and to be thinner at baseline compared to the original EPIC sample (data not shown). Selection bias may affect the observed effect estimates if the associations between diet and visceral adiposity were different in less healthy populations, i.e. sedentary or obese individuals; however, no evidence of effect modification by physical activity level or baseline BMI was observed, suggesting that selection bias are unlikely to affect these results.

The effect of the macronutrient composition of the diet on abdominal and/or visceral fat stores is controversial [32], [33]. In the present study none of the major macronutrients (protein, fat, or carbohydrate) were associated with ΔWC_BMI_. Alcohol intake was modestly associated with ΔWC_BMI_ among women. Similar results have been observed in previous prospective studies, in which high alcohol intake, especially from beer and spirits, was associated with an increase in waist circumference in women, while in men results were more mixed [29], [34], [35], [36], [37], [38]. Reason for such divergent effect in men and women are difficult to elucidate, and could be related to the specific effect of different alcohol subtypes, or to the effect of the alcohol drinking pattern.

ED has been speculated to be a key element in body-weight regulation because it may alter appetite control signals (i.e. hunger and satiety). Despite the fact that the suggested mechanism linking higher ED diets with adiposity is through increasing total energy intake and body weight, results of this study and previous DiOGenes studies [17] suggest that the effect of ED on abdominal and visceral adiposity seems to be independent of total energy intake and weight gain. Nevertheless, others authors have observed different results [39]. More research is needed to clarify whether ED has a specific effect of visceral fat accumulation.

In agreement with the results of the present study, aspects of carbohydrate quality, such as fibre, GI and GL are among the dietary factors that have been more constantly associated with less abdominal and/or visceral adiposity [27], [28], [40], [41], [42]. Fibre and GI/GL share some common mechanism by which they may affect abdominal fat accumulation. The mild blood glucose and insulin response following a high fibre/low GI diet could stimulate a higher satiation and satiety, thus leading to a decrease in energy intake and consequent weight gain. In addition, it has been hypothesized that the postprandial glucose and insulin response can affect nutrient partitioning in a way that encourages body fat storage, and that the visceral fat may be more susceptible to the influence of high insulin responses as compared to subcutaneous fat [28]. In the present study, high GI diets were associated with an increase in visceral adiposity in both men and women, but the effects of GL and fibre on visceral adiposity were observed in women only. It was explored whether the null results observed in men were due to over-adjustment (running less adjusted models) and whether the effect of fibre and GL on men's visceral adiposity were present only at very high levels of intake (comparing the highest quintile of intake to the lowest); however, no significant results were observed.

Interestingly, despite of no evidence of centre heterogeneity, the association between GI/GL and ΔWC_BMI_ was null in Florence (Italy) (data not shown). This result is similar to that of a previous study conducted in Spain, in which the lack of association between GI/GL and obesity was in part attributed to underlying dietary patterns [43]. They hypothesized that in Mediterranean populations, dietary patterns rich in cereal products, fruit, vegetables and legumes (i.e. Mediterranean diet) may lead to high GL diets, and hence the effect of GI/GL on obesity may be modified by the underlying diet. In EPIC, Italian men and women are among those with highest mean GL values, and similarly, cereal & cereal products and fruits are the major contributors to GL in this population [44].

Taken all these results together, we can predict that a man or a woman with a low ED diet and low GI diet (i.e. those simultaneously within the 1^st^ tertile of ED (<1.5 kcal/g) and the 1^st^ tertile of GI (<55 units); ≅16% of the total sample) will gain around 1.2 cm less WC than expected for a given gain in BMI in 10 years, compared to those with a high ED (>1.7 kcal/g) and GI (>59 units) diets (≅16% of the total sample). Future research is needed to determine to what extend this less WC_BMI_ gain translates into a reduction in visceral fat depot accumulation, as well as into a lower risk of developing chronic diseases.

In conclusion, results of this study suggest that in both women and men a diet with high GI and high ED may promote visceral adiposity, defined as the prospective changes in the phenotype WC_BMI_. Additional effects may be obtained among women of high alcohol, high GL, and low fibre intake.

## Supporting Information

Figure S1 Association between ED and changes in WCbmi. The values presented are regression coefficients (95% CIs) representing the annual change in waist circumference for a given body mass index (DeltaWCbmi, cm/y) for 1 kcal/g increase in energy density (ED) in men (A) and women (B). Models were adjusted for age, baseline weight, baseline height, baseline WCbmi, smoking, alcohol intake, physical activity, education, follow-up duration, energy from drinks, menopausal status (women only), and hormone replacement therapy use (women only). Overall estimates were made on the basis of random-effect models. Number of participants per study centre: Florence (1,141 men and 3,940 women); Norfolk (2,626 men and 3,640 women); Amsterdam/Maastricht (1,507 men and 2,026 women); Doetinchem (1,419 men and 1,525 women); Potsdam (3,042 men and 5,619 women); Copenhagen/Aarhus (9,959 men and 12,187 women).
(0.06 MB DOC)

Figure S2 Association between GI and changes in WCbmi. The values presented are regression coefficients (95% CIs) representing the annual change in waist circumference for a given body mass index (deltaWCbmi, cm/y) for 10 units increase in glycemic index (GI) in men (A) and women (B). Models were adjusted for age, baseline weight, baseline height, baseline WCbmi, smoking, alcohol intake, physical activity, education, follow-up duration, fibre, carbohydrate, fat and protein, menopausal status (women only), and hormone replacement therapy use (women only). Overall estimates were made on the basis of random-effect models. Number of participants per study centre: Florence (1,141 men and 3,940 women); Norfolk (2,626 men and 3,640 women); Amsterdam/Maastricht (1,507 men and 2,026 women); Doetinchem (1,419 men and 1,525 women); Potsdam (3,042 men and 5,619 women); Copenhagen/Aarhus (9,959 men and 12,187 women).
(0.06 MB DOC)
