# Supplementary material for: Dietary Determinants of Changes in Waist Circumference Adjusted for Body Mass Index – a Proxy Measure of Visceral Adiposity
Source: PLoS One. 2010 Jul 14;5(7):e11588. doi: 10.1371/journal.pone.0011588 (PMC2904387; doi:10.1371/journal.pone.0011588)
Supplement: Figure S1 — Association between ED and changes in WCbmi. The values presented are regression coefficients (95% CIs) representing the annual change in waist circumference for a given body mass index (DeltaWCbmi, cm/y) for 1 kcal/g increase in energy density (ED) in men (A) and women (B). Models were adjusted for age, baseline weight, baseline height, baseline WCbmi, smoking, alcohol intake, physical activity, education, follow-up duration, energy from drinks, menopausal status (women only), and hormone replacement therapy use (women only). Overall estimates were made on the basis of random-effect models. Number of participants per study centre: Florence (1,141 men and 3,940 women); Norfolk (2,626 men and 3,640 women); Amsterdam/Maastricht (1,507 men and 2,026 women); Doetinchem (1,419 men and 1,525 women); Potsdam (3,042 men and 5,619 women); Copenhagen/Aarhus (9,959 men and 12,187 women). (0.06 MB DOC) [file pone.0011588.s001.doc]

Overall (I-squared = 0.0%, p = 0.531)

Potsdam (Germany)

Amsterdam / Maastricht (NL)

Copenhagen /Aarhus (Denmark)

Norfolk (UK)

Study Centre

Doetinchem (NL)

Florence (Italy)

0.09 (0.05, 0.13)

0.12 (0.05, 0.19)

0.01 (-0.11, 0.13)

0.12 (0.04, 0.19)

0.05 (-0.08, 0.18)

ΔWC_BMI_ (cm/y) (95% CI)

0.08 (-0.06, 0.23)

0.02 (-0.11, 0.15)

0

-.4

-.3

-.2

-.1

.1

.2

.3

.4

**Figure S1**

**(A)**

Overall (I-squared = 58.4%, p = 0.035)

Potsdam (Germany)

Doetinchem (NL)

Copenhagen /Aarhus (Denmark)

Study Centre

Norfolk (UK)

Florence (Italy)

Amsterdam / Maastricht (NL)

0.15 (0.09, 0.21)

0.11 (0.05, 0.16)

-0.04 (-0.26, 0.17)

0.26 (0.18, 0.35)

ΔWC_BMI_ (cm/y) (95% CI)

0.15 (0.01, 0.28)

0.16 (0.09, 0.23)

0.13 (0.04, 0.23)

0

-.4

-.3

-.2

-.1

.1

.2

.3

.4

**(B)**
