# Supplementary material for: Dietary Determinants of Changes in Waist Circumference Adjusted for Body Mass Index – a Proxy Measure of Visceral Adiposity
Source: PLoS One. 2010 Jul 14;5(7):e11588. doi: 10.1371/journal.pone.0011588 (PMC2904387; doi:10.1371/journal.pone.0011588)
Supplement: Figure S2 — Association between GI and changes in WCbmi. The values presented are regression coefficients (95% CIs) representing the annual change in waist circumference for a given body mass index (deltaWCbmi, cm/y) for 10 units increase in glycemic index (GI) in men (A) and women (B). Models were adjusted for age, baseline weight, baseline height, baseline WCbmi, smoking, alcohol intake, physical activity, education, follow-up duration, fibre, carbohydrate, fat and protein, menopausal status (women only), and hormone replacement therapy use (women only). Overall estimates were made on the basis of random-effect models. Number of participants per study centre: Florence (1,141 men and 3,940 women); Norfolk (2,626 men and 3,640 women); Amsterdam/Maastricht (1,507 men and 2,026 women); Doetinchem (1,419 men and 1,525 women); Potsdam (3,042 men and 5,619 women); Copenhagen/Aarhus (9,959 men and 12,187 women). (0.06 MB DOC) [file pone.0011588.s002.doc]

Overall (I-squared = 42.5%, p = 0.122)

Study Centre

Norfolk (UK)

Copenhagen /Aarhus (Denmark)

Doetinchem (NL)

Amsterdam / Maastricht (NL)

Potsdam (Germany)

Florence (Italy)

0.07 (0.03, 0.12)

ΔWC_BMI_ (cm/y) (95% CI)

0.20 (0.06, 0.33)

0.03 (-0.02, 0.08)

0.08 (-0.04, 0.19)

0.10 (0.01, 0.20)

0.09 (0.03, 0.16)

-0.00 (-0.10, 0.10)

0

-.4

-.3

-.2

-.1

.1

.2

.3

.4

**Figure S2**

**(A)**

Overall (I-squared = 35.1%, p = 0.173)

Study Centre

Potsdam (Germany)

Norfolk (UK)

Doetinchem (NL)

Florence (Italy)

Amsterdam / Maastricht (NL)

Copenhagen /Aarhus (Denmark)

0.06 (0.03, 0.10)

ΔWC_BMI_ (cm/y) (95% CI)

0.10 (0.05, 0.14)

-0.05 (-0.18, 0.08)

0.07 (-0.09, 0.23)

0.02 (-0.03, 0.08)

0.09 (0.01, 0.16)

0.09 (0.02, 0.15)

0

-.4

-.3

-.2

-.1

.1

.2

.3

.4

**(B)**
